# Poor sleep health is associated with worse cardiometabolic risk among rural and urban schooled Nigerian adolescents

**DOI:** 10.1038/s41598-025-25659-0

**Published:** 2025-11-25

**Authors:** Oluwatosin Eunice Olorunmoteni, Karine Scheuermaier, Adesegun Olayiwola Fatusi, Joan Ifeoluwa Akande, Francesc Xavier Gómez-Olivé

**Affiliations:** 1 Department of Paediatrics and Child Health, College of Health Sciences, Obafemi Awolowo University, Ile-Ife, Osun State Nigeria; 2 Wits Sleep Laboratory, Brain Function Research Group, School of Physiology, Faculty of Health Sciences, University of the Witwatersrand, Johannesburg, South Africa; 3 School of Public Health, University of Medical Sciences, Ondo, Ondo State Nigeria; 4 Department of Community Health, College of Health Sciences, Obafemi Awolowo University, Ile-Ife, Osun State Nigeria; 5 Department of Paediatrics, Obafemi Awolowo University Teaching Hospitals Complex, Ile-Ife, Osun State Nigeria; 6 MRC/Wits Rural Public Health and Health Transitions Research Unit (Agincourt), School of Public Health, Faculty of Health Sciences, University of the Witwatersrand, Johannesburg, South Africa

**Keywords:** Sleep characteristics, Sleep disorders, Metabolic disease, Cardiovascular disease, Cardiometabolic disease, Neuroscience, Neurology

## Abstract

There is limited evidence on the association between sleep health and cardio-metabolic risk (CMR) in Sub-Saharan African adolescents. Using a cross-sectional design, we assessed the association between sleep health and CMR, including 900 adolescents aged 13–19, attending six rural and six urban schools, in Osun State, Nigeria. Sleep health variables included sleep quality and duration (Pittsburgh Sleep Quality Index), risk of sleep apnea (Teen STOPBANG), and daytime sleepiness (Epworth Sleepiness Scale for Children and Adolescents). A continuous CMR-score was calculated using z-scores of mean arterial pressure (MAP), body mass index (BMI), waist circumference (WC), and random blood glucose. We analysed the association between sleep health, place of residence, and CMR using generalized linear models. Urban adolescents presented worse sleep health indicators and higher BMI, WC, and CMR-score (*p* < 0.05). Overall, poorer sleep quality was associated with higher CMR-score (β = 0.02), BMI z-score (β = 0.04), and WC z-score (β = 0.03) (*p* < 0.05), and lower sleep duration with higher CMR-score (β = 0.04), BMI z-score (β = 0.11), and WC z-score (β = 0.07) (*p* < 0.05). Risk of sleep apnea was associated with higher CMR-score (β = 0.08), MAP z-score (β = 0.17), and WC z-score (β = 0.14) (*p* < 0.05). Improving the sleep health of adolescents may prevent cardiometabolic diseases later in life.

**Supplementary Information:**

The online version contains supplementary material available at 10.1038/s41598-025-25659-0.

**Introduction**.

Sleep is vital for human health and function^1,2^. Sleep is judged to be healthy if it is appropriate in duration, quality, and timing and is associated with sustained alertness in waking hours^1,3^. Hence, the association between sleep and health is better assessed using multiple dimensions of sleep. However, the available evidence for an association between poor sleep health and adverse health outcomes has largely focused on sleep duration and sleep quality^4,5^.

The emergence of the concept of sleep health has widened the understanding of sleep as being multidimensional. Global sleep researchers have identified that it may be critical to assess the various dimensions of sleep health^1,2,6^. Sleep health has been defined by Buysse as “a multidimensional pattern of sleep-wakefulness, adapted to individual, social, and environmental demands, that promotes physical and mental well-being^1^.” Studying the multiple dimensions of sleep health is very important when investigating the association between sleep and cardiometabolic health^2^. However, the association of the multiple dimensions of sleep health with CMR needs to be studied further^5,7^.

In Africa, sleep is an important but poorly studied aspect of health. Individuals of African ancestry have been reported to have a higher prevalence of extreme sleep duration (short and long), higher prevalence of sleep-disordered breathing, insomnia, and cardiovascular diseases^8,9^. Among individuals of African ancestry, sleep problems have been attributed to genetic causes, infections, immunological, cultural, as well as environmental factors^8,9^. Furthermore, a few studies have reported an association between adolescent sleep health and measures of adiposity, such as body mass index, in an African setting (Uganda)^10,11^. Therefore, the association between the different dimensions of adolescent sleep health and the development of CMR needs more in-depth study in the African setting.

Among adolescents, insufficient sleep duration and /or poor quality sleep have been associated with increased cardiometabolic risk (CMR), including hypertension, obesity, and diabetes^12,13^. However, the few available studies on the association between sleep and cardiometabolic risk in Sub-Saharan African adolescents were focused on the association between sleep duration and body mass index (BMI), with some consideration of the effect of physical inactivity, although the findings were conflicting^14,15^. In Nigeria, sleep problems among adolescents include disorders of quality and quantity of sleep^16–19^. However, Nigerian studies have focused on obesity as a contributor to higher CMR^20^, while other CMR outcomes (glucose, hypertension, etc.) remain poorly studied. In addition, the contribution of other sleep variables to CMR (such as sleep quality, sleep apnea and daytime sleepiness) has not been well explored^4^. Thus, there is a need to understand the mechanisms by which different dimensions of sleep health contribute to the cardiometabolic risk profile of adolescents in an African setting.

Disparities in sleep health among rural and urban adolescents may be linked to CMR in different ways. In a recently published white paper by the Sleep Research Society^21^, one of the gaps identified as requiring urgent attention is the need to study sleep health in children and adolescents by exploring sleep disparities^21^. In Nigeria, there is a wide rural-urban social disparity with rural areas having much less electricity at night than urban areas^22,23^. This may result in higher light exposure at night in urban adolescents. Evening light exposure has a known association with delayed bedtimes and poorer sleep^24^. We have recently shown that, indeed, urban adolescents in Nigeria have worse sleep health and more delayed bedtimes than rural adolescents^25^. Urban areas in Nigeria have also gone through lifestyle changes characterised by reduced physical activity and more sedentary lifestyles with higher access to high-carbohydrate/high-fat diets than in rural areas, which may contribute independently to higher CMR^20^. Thus, there is a need to compare sleep health and its association with CMR between urban-based and rural-based adolescents.

Hence, this study’s primary objective was to investigate the association between the sleep health of adolescents and markers of CMR in rural versus urban in-school adolescents. We hypothesised that urban adolescents had higher CMR than rural adolescents and that poor sleep health indicators were associated with a higher CMR among Nigerian in-school adolescents.

## Results

In total, 900 adolescents (459 rural; 59% female; average age 15.1 ± 1.4; 49.1% of lower social class) were included in this study. More detailed background demographic information can be found in the methods section below (Background demographic information).

### Sleep health of the adolescents

The rural and urban distribution of the sleep health variables of the adolescents is shown in Table 1. All markers of sleep health were worse in urban compared to rural adolescents. Sleep quality was lower (*p* < 0.001) (PSQI score higher), sleep duration was lower (*p* < 0.001), and daytime sleepiness was higher (*p* = 0.033) in the urban adolescents when compared to the rural adolescents. Similarly, urban adolescents had a higher prevalence of risk of sleep apnea than rural adolescents (*p* = 0.006), as measured by having 3 components or more present out of 8 possible components of the Teen STOPBANG. The average number of components of the Teen STOPBANG present was higher in the urban compared to the rural adolescents (unpaired t-test, *p* < 0.001).

**Table 1:** Comparison of the participant characteristics, sleep and cardio-metabolic risk variables between rural and urban-dwelling adolescents.

Participant characteristics	Whole Population Mean ± SD Or *N* (%)	Rural Mean ± SD Or *N* (%)	Urban Mean ± SD Or *N* (%)	*p*-value
Gender				0.665*
Female	531 (59.0)	274 (59.7)	257 (58.3)	
Male	369 (41.0)	185 (40.3)	184 (41.7)	
Age in years				**< 0.001**
Mean ± SD	15.1 ± 1.4	15.4 ± 1.4	14.8 ± 1.4	
Parental history of cardiometabolic disease				
Absent	857 (95.2)	449 (97.8)	408 (92.5)	< 0.001*
Present	43 (4.8)	10 (2.2)	33 (7.5)	
Physical activity level (*N* = 858)				
Low	210 (24.5)	108 (24.9)	102 (24.0)	0.195*
Moderate	489 (57.0)	255 (58.9)	234 (55.1)	
Vigorous	159 (18.5)	70 (16.2)	89 (20.1)	
Sleep health variables				
Sleep quality (PSQI)	2.7 ± 2.6	2.3 ± 2.4	3.1 ± 2.8	**< 0.001**
Daytime sleepiness score (ESS)	5.3 ± 3.9	5.0 ± 3.8	5.5 ± 4.0	**0.033**
Risk of sleep apnea^¥^				
Low risk	805 (90.2)	422 (92.9)	383 (87.4)	0.006*
Risk of apnea	87 (9.8)	32 (7.1)	55 (12.6)	
Teen STOPBANG continuous (0–8)	1.20 ± 0.98	1.08 ± 0.93	1.32 ± 1.01	**< 0.001**
Sleep Duration (h: mm)	7.17 ± 1.67	7.38 ± 1.57	6.92 ± 1.73	**< 0.001**
Cardiometabolic risk variables				
CMR score	-0.2 ± 0.6	-0.2 ± 0.5	-0.1 ± 0.7	0.392
Weight (kg)	48.4 ± 9.5	46.5 ± 7.8	50.3 ± 10.7	**< 0.001**
Height (m)	1.59 ± 0.1	1.57 ± 0.1	1.61 ± 0.1	**< 0.001**
BMI in kg/m^2^	18.9 ± 2.8	18.7 ± 2.3	19.2 ± 3.3	**0.004**
BMI z-score	-0.6 ± 1.0	-0.7 ± 0.9	-0.5 ± 1.2	**< 0.001**
Waist Circumference (cm)	68.9 ± 6.5	68.1 ± 5.5	69.6 ± 7.3	**0.001**
WC z-score	0.0	-0.1 ± 0.9	0.1 ± 1.1	**< 0.001**
Neck Circumference (cm)	30.1 ± 4.0	29.7 ± 4.4	30.5 ± 3.6	**0.007**
NC z-score	0.00	-0.2 ± 0.9	0.2 ± 1.0	**< 0.001**
SBP in mmHg	113.5 ± 12.1	113.2 ± 11.7	113.7 ± 12.5	0.453
DBP in mmHg	69.9 ± 9.2	69.6 ± 9.0	70.2 ± 9.4	0.398
MAP in mmHg	84.4 ± 9.0	84.1 ± 8.7	84.7 ± 9.3	0.360
MAP z-score	0.0 ± 1.0	-0.1 ± 1.0	0.1 ± 1.0	0.360
Random blood glucose (RBG) (mmol/L)	7.3 ± 1.9	7.72.1	6.8 ± 1.7	**< 0.001**
RBG z score	0.0 ± 1.0	0.2 ± 1.1	− 0.2 ± 0.9	**<0.001**

*PSQI* Pittsburgh Sleep Quality Index, *ESS* Epworth Sleepiness Scale, *BMI* body mass index, *SBP* systolic blood pressure, *DBP* diastolic blood pressure, *NC* neck circumference, *WC* waist circumference, *MAP* Mean arterial pressure, *CMR* cardiometabolic risk, *RBG* random blood glucose.

^¥^Low risk: presence of < 3 out of 8 components of the Teen STOPBANG. Risk of sleep apnea: presence of ≥ 3 components out of 8 of the Teen STOPBANG.

*Chi-square test. Other analyses were with independent t-tests.

### Cardiometabolic risk outcomes

BMI (*p* = 0.004), WC (*p* < 0.001), and neck circumference (NC) (*p* = 0.007) were significantly higher in the urban compared to rural adolescents. Conversely, random blood glucose (RBG) was higher among the rural compared to urban adolescents (*p* < 0.001). There was no statistically significant difference between the systolic blood pressure (SBP), diastolic blood pressure (DBP), and MAP of the urban versus rural adolescents, as shown in Table 1.

### Relationship between sleep health and cardiometabolic risk outcomes of the adolescents

There was a statistically significant association between higher CMR and lower sleep quality (higher PSQI score) lower sleep duration, and risk of sleep apnea, using the raw values of the cardiometabolic risk measures (Table 2). Using the simple linear regression (unadjusted), lower sleep quality (β = 0.02, *p* < 0.001) and Teen STOPBANG continuous score (β = 0.08, *p* < 0.001) were associated with higher CMR scores. Lower sleep duration was associated with a higher CMR score (β=-0.04, *p* = 0.001) (Table 3). Lower sleep quality (higher PSQI score) was associated with a higher BMI (*p* = 0.001), WC (*p* = 0.018), and NC z-scores (*p* < 0.001). Higher Teen STOPBANG continuous score was associated with a higher BMI (*p* = 0.028), SBP (*p* < 0.001), and DBP (*p* = 0.001); and higher WC, NC, and MAP z-scores (*p* = 0.001). Longer sleep duration was associated with a lower BMI (*p* < 0.001), WC (*p* = 0.001), and NC (all *p* < 0.001). There was no statistically significant relationship between daytime sleepiness and any of the CMR variables measured.

**Table 2:** Bivariate relationship of sleep characteristics with cardiometabolic risk factors and neck circumference (non-z-scores).

	BMI	*P*-value	WC	*P*-value	NC	*P*-value	SBP	*P*-value	DBP	*P*-value	RBG	*P*-value
	Beta estimate (crude)		Beta estimate (crude)		Beta estimate (crude)		Beta estimate (crude)		Beta estimate (crude)		Beta estimate (crude)	
	95%CI		95%CI		95%CI		95%CI		95%CI		95%CI	
Sleep quality^α^	0.13	**< 0.001** ^*^	0.24	< **0.001**	0.11	**0.003**	0.11	0.469	-0.01		-0.02	
	0.06, 0.20		0.13, 0.36		0.04, 0.18		-0.19, 0.42		-0.24, 0.22	0.929	-0.08, 0.02	0.290
Daytime sleepiness^β^	-0.01	0.604	0.01	0.91	0.02	0.568	0.04	0.732	-0.14		-0.01	
	-0.06, 0.04		-0.10, 0.12		-0.05, 0.09		-0.17, 0.24		-0.29, 0.02	0.084	-0.05, 0.02	0.444
Teen STOPBANG continuous	0.21	**0.028**	0.55	**0.015**	0.7	< **0.001**	2.58	**< 0.001**	1.04		-0.06	
	0.02, 0.40		0.11, 0.99		0.52, 0.88		1.79, 3.38		0.43, 1.65	**0.001**	-0.19, 0.07	0.373
Sleep duration^γ^	-0.31	**< 0.001**	-0.47	< **0.001**	-0.26	**0.001**	-0.38	0.122	-0.01		0.04	
	-0.42, -0.20		-0.72, -0.21		-0.42, -0.11		-0.85, 0.10		-0.37, 0.35	0.960	-0.04, 0.11	0.338

*BMI* Body Mass Index in (kg/m^2^), *WC* waist circumference in cm, *NC* neck circumference in cm, *SBP* systolic blood pressure in mmHg, *DBP* diastolic blood pressure in mmHg, *RBG* random blood glucose. ^α^Based on the Global PSQI score: higher scores indicate lower sleep quality. ^β^Measured with the Epworth Sleepiness Scale – higher scores indicate higher daytime sleepiness. ^γ^As determined by the sleep duration question on the PSQI.

**Table 3:** Bivariate relationship between sleep characteristics and cardiometabolic risk factors (z-scores).

	CMR *Score* Beta estimate (crude) 95%CI	*P*-value	BMI *z-score* Beta estimate (crude) 95%CI	*P*-value	WC *z-score* Beta estimate (crude) 95%CI	*P*-value	NC *z-score* Beta estimate (crude) 95%CI	*P*-value	MAP *z-score* Beta estimate (crude) 95%CI	*P*-value	RBG *z-score* Beta estimate (crude) 95%CI	*P*-value
Sleep quality^α^	0.02	**0.034**	0.04	**0.001**	0.03	**0.018**	0.05	**< 0.001**	0.01	0.790	-0.01	0.257
	0.01, 0.03		0.02, 0.07		0.01, 0.06		0.02, 0.07		-0.02, 0.03		-0.04, 0.01	
Daytime sleepiness^β^	-0.01	0.490	0.01	0.869	-0.01	0.927	0.01	0.791	-0.01	0.305	0.01	0.444
	-0.01, 0.01		-0.02, 0.02		-0.18, 0.16		-0.01, 0.02		-0.03, -0.01		0.02, 0.01	
Teen STOP BANG continuous	0.08	< **0.001**	0.04	0.246	0.14	**< 0.001**	0.18	< **0.001**	0.17	**< 0.001**	**-**0.03	0.373
	0.04, 0.12		-0.03, 0.11		0.07, 0.21		0.11, 0.25		0.11, 0.24		-0.10, 0.04	
Sleep duration (h)^γ^	-0.04	**0.001**	-0.11	**< 0.001**	-0.65	**0.001**	-0.08	**< 0.001**	-0.15	0.465	0.02	0.338
	-0.07, -0.02		-0.14, -0.06		-0.10, -0.03		-0.11, -0.04		-0.05,-0.02		-0.02, 0.06	

*CMR* cardiometabolic risk, *BMI* body mass index, *WC* waist circumference, *NC* neck circumference, *MAP* mean arterial blood pressure, *RBG* random blood glucose.

^α^Based on the Global PSQI score: higher scores indicate lower sleep quality.

^β^Measured with the Epworth Sleepiness Scale – higher scores indicate higher daytime sleepiness.

^γ^As determined by the sleep duration question on the PSQI.

In the multivariable linear regression models adjusting for parental history of cardiometabolic disease and physical activity, when comparing the urban to the rural adolescents, BMI, WC, and NC were all higher, while random blood glucose (RBG) was significantly lower; and there was no difference in blood pressure measurements and CMR score. In these adjusted models, we found the following associations of sleep health variables with CMR variables. Lower sleep quality (higher PSQI score) was associated with a higher CMR score (β = 0.18), BMI z-score (β = 0.04), WC z-score (β = 0.03), and NC z-score (β = 0.04). A higher Teen STOPBANG continuous score was associated with a higher CMR score (β = 0.09), MAP z-score (β = 0.2), WC z-score (β = 0.14), and NC z-score (β = 0.18). Conversely, longer sleep duration was associated with lower CMR score (β= -0.04), BMI z-score (β= -0.09), WC z-score (β= -0.06), and NC z-score (β= -0.07) (Table 4).

**Table 4:** Multivariable linear regression models assessing the relationship between sleep health variables (independent variable) and cardiometabolic risk factors as z-scores (dependent variable).

	Dependent variable											
Independent variables	CMR Score Beta estimate (adjusted) 95%CI	*P-value*	BMI z-score Beta estimate (adjusted) 95%CI	*P-value*	WC z-score Beta estimate (adjusted) 95%CI	*P-value*	NC z-score Beta estimate (adjusted) 95%CI	***P-value***	MAPz**-scoreBeta estimate (adjusted) 95%CI**	***P-value***	**RBG** **z-score** **Beta estimate (adjusted) 95%CI**	***P-value***
**Sleep quality** ^**α**^	0.18	**0.026**	0.04	**0.001**	0.03	< **0.001**	0.04	**0.001**	-0.01	0.944	-0.01	0.702
	0.01, 0.03		0.02, 0.07		0.02, 0.05		0.02, 0.07		-0.03, 0.03		-0.03, 0.02	
Residence (urban-rural)*	0.03	0.545	0.23	**0.001**	0.23	**0.001**	0.41	**< 0.001**	0.06	0.395	-0.44	**< 0.001**
	-0.06, 0.11		0.09, 0.37		0.09, 0.37		0.28, 0.55		-0.08, 0.20		-0.57, -0.31	
Parental history of CMR^†^	0.15	0.128	0.30	0.069	-0.01	0.830	-0.18	0.242	0.25	0.119	0.03	0.855
	-0.04, 0.35		-0.02, 0.61		-0.32, 0.32		-0.49, 0.12		-0.06,0.57		-0.28, 0.33	
Physical activity^¥^	-0.07	**0.024**	-0.25	**< 0.001**	-0.02	0.715	-0.10	0.064	-0.07	0.208	0.05	0.332
	-0.14, -0.01		-0.36, -0.15		-0.12, 0.08		-0.20, 0.07		-0.17, 0.04		-0.05, 0.15	
Daytime sleepiness ^**β**^	-0.01	0.456	0.01	0.988	-0.01	0.911	-0.01	0.941	-0.01	0.118	-0.01	0.873
	-0.01, 0.01		-0.02, 0.02		-0.02, 0.02		-0.02, 0.02		-0.03, 0.01		-0.02, 0.02	
Residence (urban-rural)*	0.04	0.351	0.26	< **0.001**	0.27	**< 0.001**	0.44	< **0.001**	0.07	0.311	-0.45	< **0.001**
	-0.04, 0.12		0.12, 0.40		0.12, 0.40		0.31, 0.58		-0.07, 0.21		-0.58, -0.32	
Parental history of CMR^†^	0.17	0.082	0.34	**0.038**	0.026	0.872	-0.14	0.370	0.26	0.102	0.02	0.873
	-0.02, 0.37		0.02, 0.66		-0.29, 0.35		-0.45, 0.17		0.05, 0.58		-0.28, 0.32	
Physical activity^¥^	-0.07	**0.036**	-0.24	**< 0.001**	-0.01	0.792	-0.08	0.115	-0.07	0.199	0.05	0.345
	-0.13, -0.01		-0.35, 0.14		-0.12, 0.09		-0.18, 0.02		-0.17, 0.04		-0.05, 0.15	
**Teen STOPBANG continuous**	0.09	< **0.001**	0.05	0.176	0.14	< **0.001**	0.18	< **0.001**	0.20	< **0.001**	-0.01	0.674
	0.05, 0.14		-0.02, 0.12		0.07, 0.21		0.11, 0.25		0.13, 0.27		-0.08, 0.05	
Residence (urban-rural)*	0.01	0.790	0.23	**< 0.001**	0.23	**0.001**	0.40	< **0.001**	0.01	0.970	-0.44	< **0.001**
	-0.07, 0.10		0.11, 0.39		0.10, 0.37		0.27, 0.53		-0.14, 0.13		-0.57, -0.31	
Parental history of CMR^†^	0.16	0.113	0.33	**0.043**	0.01	0.979	-0.16	0.285	0.22	0.160	0.03	0.860
	-0.04, 0.35		0.01, 0.65		-0.31, 0.32		-0.47, 0.14		-0.09, 0.53		-0.27, 0.33	
Physical activity^¥^	-0.09	**0.008**	-0.26	< **0.001**	-0.04	0.434	-0.12	**0.017**	-0.10	0.047	0.05	0.287
	-0.15, -0.02		-0.36, -0.15		-0.15, 0.06		-0.22, -0.02		-0.21, -0.01		-0.05, 0.15	
**Sleep duration (h)** ^**γ**^	-0.04	**0.002**	-0.09	< **0.001**	-0.06	**0.004**	-0.07	**0.001**	-0.01	0.686	0.01	0.839
	-0.65, -0.02		-0.14, -0.05		-0.10, -0.02		-0.11, -0.03		-0.05, 0.03		-0.04, 0.04	
Residence (urban-rural)*	0.02	0.624	0.22	**0.002**	0.24	**0.001**	0.42	< **0.001**	0.06	0.391	-0.45	< **0.001**
	-0.07, 0.11		0.08, 0.35		0.10, 0.38		0.28, 0.55		-0.08, 0.20		-0.58, -0.31	
Parental history of CMR^†^	0.15	0.133	0.29	0.072	-0.01	0.977	-0.18	0.261	0.24	0.134	0.03	0.870
	-0.05, 0.34		-0.03, 0.61		-0.32, 0.31		-0.48, 0.13		-0.07, 0.56		-0.28, 0.33	
Physical activity^¥^	-0.07	**0.028**	-0.25	< **0.001**	-0.02	0.730	-0.09	0.092	-0.07	0.203	0.05	0.341
	-0.13, -0.01		-0.35, -0.15		-0.12, 0.09		-0.19, 0.01		-0.17, 0.04		-0.05, 0.15	

*CMR* cardiometabolic risk, *BMI* body mass index, *WC* waist circumference, *NC* neck circumference, *MAP* mean arterial blood pressure, *RBG* random blood glucose.

*Reference group = rural †reference group = no risk.

^¥^Physical activity was entered as an ordinal categorical variable coded 0 (low PA), 1 (moderate PA) or 2 (high PA) with an assumption of linearity between PA and the cardiometabolic outcome variables.

^α^Based on the Global PSQI score: higher scores indicate lower sleep quality.

^β^Measured with the Epworth Sleepiness Scale – higher scores indicate higher daytime sleepiness. ^γ^ As determined by the sleep duration question on the PSQI.

## Discussion

### Key findings

In this cross-sectional study of 900 Nigerian in-school adolescents, urban compared to rural dwellings, and poorer sleep health were associated with higher cardiometabolic risk. Urban adolescents had higher BMI, WC, and NC while rural-dwelling adolescents had higher random blood glucose levels; the CMR score combining z-scores of the 4 clinical CMR factors (BMI, WC, RBG, and MAP) was not different between the urban and rural adolescents. Urban adolescents also had poorer sleep health measures than their rural counterparts. Overall, lower sleep quality was associated with higher BMI, WC, and NC, and a higher CMR score. A higher risk of sleep apnea was associated with higher CMR scores, and higher WC, NC, and MAP z-scores. Higher sleep duration was associated with lower CMR scores, and lower BMI, WC, and NC z-scores.

### Rural-urban difference in sleep health and cardiometabolic risk

Our study is unique among earlier studies on the association between sleep and cardiometabolic risk due to its comparison of an urban and a rural population of adolescents in a low and middle-income country. This study highlights the important contribution of urban versus rural type of residence to the relationship between sleep health and cardiometabolic health in line with previous publications^2,8^. Among the social determinants of sleep health, racial and neighbourhood factors are important contributors to the sleep health of adolescents^2,26^. Our study shows how the urban lifestyle may contribute independently to both poorer cardiometabolic health and poorer sleep health, respectively. In addition, poorer sleep health was independently associated with increased cardiometabolic risk.

The higher cardiometabolic risk in urban adolescents concerned anthropometric measures of adiposity (WC, NC, BMI). This finding may be associated with dietary lifestyles in urban areas (higher availability of high sugar, high fat processed foods)^31^. In contrast, the RBG of rural adolescents in our study was higher than that of urban adolescents. We excluded adolescents with known diabetes from our study. It is possible that the lower access to healthcare facilities in the rural communities^27^ meant a lower rate of diabetes detection, and thus we may have included, unwittingly, more rural than urban adolescents with undiagnosed pre-diabetes or diabetes. Measures of blood pressure were not significantly different between rural and urban adolescents. This finding is contrary to the report of higher SBP and DBP among urban adolescents in a study among 500 urban vs. 500 rural Nigerian adolescents in the same senatorial district as our study, though conducted ten years before our study and involved adolescents aged 10–19 years^28^. However, a higher level of adiposity was reported among urban adolescents in their study, similar to the findings from our study.

### Association between the multidimensions of sleep health and CMR

We utilised multiple dimensions of sleep health in assessing the association between sleep health and CMR. We found poorer sleep health overall in the urban compared to the rural adolescents. Hypotheses for poorer sleep health in urban adolescents include higher access to electricity and thus light at night (which may disrupt circadian rhythms) and overall poorer sleep habits, which may be multifactorial. The industrialised lifestyle in urban communities has been shown to predispose adolescents to greater access to screens and bedtime screen use (driven by larger night access to electricity compared to rural areas). This, in turn, leads to higher levels of artificial light at night (ALAN), which causes circadian rhythm disruption and delayed bedtimes^24,29^. The delayed bedtimes, while school start times and thus wake times cannot be delayed, would lead to the lower sleep duration observed in urban adolescents, but also may create a chronic circadian misalignment resulting in the poorer sleep quality observed. Furthermore, there is a possible higher exposure to sleep disruptors in urban settings, in particular, noise. It has also been suggested that reduced parental supervision, due to busy urban lifestyles, is another possible explanation for urban adolescents’ poorer sleep^20–22^.

### Sleep quality and CMR

The first marker of sleep health that we studied was overall sleep quality measured by the PSQI global score. Urban adolescents reported poorer sleep quality than rural adolescents. Poorer sleep quality was associated with higher CMR scores and higher measures of adiposity (BMI, WC, and NC) among the study adolescents. This suggests that poorer quality sleep may contribute to worse cardiometabolic health in adolescents. The association between lower sleep quality and higher CMR (including measures of central adiposity like WC and BMI) has been discussed in previous research works^7,12^. In a US adolescents’ cross-sectional study by Feliciano et al., poor sleep quality based on actigraphy measurements was associated with central adiposity and an increased aggregate metabolic score^5^. Similarly, Gohil et al., in a review article, reported that poor sleep and obesity were concurrent epidemics based on the association between sleep quality, sleep duration, and obesity among adolescents^12^. Conversely, a study conducted among 1078 Chinese children and adolescents, aged 9–18, reported a lack of significant association between PSQI-derived sleep quality and CMR measured with BMI, WC, and serum lipids^7^. The inclusion of pre-adolescents and early adolescents in their study may account for this difference. Interestingly, the same study found a significant association between sleep irregularity and CMR, showing the contribution of other components of sleep health to CMR^7^. The mechanisms involved in the association between sleep quality and adiposity may be linked with the effect of sleep disruption on energy balance and the hormones regulating satiety and appetite, and a dysregulation of the hypothalamic-pituitary-adrenal axis^12^.

### Sleep duration and CMR

The second marker of sleep health measured in our study was sleep duration, as derived from the PSQI’s question on sleep duration. Urban adolescents reported shorter sleep duration than rural adolescents, while shorter sleep was associated with higher CMR. Short sleep is a well-established risk factor for adverse cardiometabolic health in adolescents^30^. The association between short sleep duration and CMR has been described in both adults^31^ and pediatric populations^5,31^, while a lack of association has also been reported^13^. In our study, shorter sleep duration was associated with higher CMR scores and higher measures of adiposity after controlling for confounders. Similar to our findings, longer sleep duration has been reported as being protective against CMR^13^, while others reported a U-shaped association, whereby both short sleep duration and extremely long sleep duration were associated with CMR in both children and adults^31^. In our study, because only eight participants had a sleep duration > 10 h (threshold) for long sleep duration in children and adolescents)^32^ We may not have had the power to detect an association between long sleep duration and CMR^25^.

### Sleep apnea and CMR

Our third measure of sleep health was risk of sleep apnea as assessed by the Teen STOPBANG. Urban adolescents had a higher risk of sleep apnea than rural adolescents, while a higher risk of sleep apnea (as shown by a higher Teen STOPBANG score) showed a significant association with a higher CMR score. We found a similar association between a higher risk of sleep apnea and measures of adiposity, specifically WC and NC. The second feature of the association between the risk of sleep apnea and CMR was the association with SBP and DBP at the bivariable level and the MAP at the multivariable level. It is noteworthy that the risk of sleep apnea was the only sleep health variable associated with MAP in our study. This may be due to the disturbances in the autonomic nervous system and endothelial dysfunction that can result from recurrent hypoxia with sleep apnea^33^. However, the low sensitivity of the Teen STOPBANG (64%) means that some adolescents with OSA could have been missed, underestimating the cardiometabolic effects of having sleep apnea. Most diagnostic tests for OSA have low sensitivity, emphasizing the need for polysomnography even in low-resource settings.

## Limitations

There are some limitations to this study. First, the cross-sectional nature of our study design does not allow for the determination of causality. Secondly, the sleep health variables were self-reported, and we could not calculate sleep regularity, another important sleep health variable, which is best determined with the use of actigraphy. Thirdly, we were unable to include more sophisticated measures of CMR, such as cardiorespiratory fitness, skinfold thickness, homeostatic model assessment of insulin resistance, and blood lipid measures, due to the existing regulations forbidding blood collection in the school setting in the study location. Our findings of a relationship between poor sleep health and higher cardiometabolic risk should be studied in other contexts. However, the strong association between sleep and CMR in this adolescent population shows the need for longitudinal research on the relationship between sleep and CMR in adolescents. Such studies will help to build on the findings from our study if the use of objective sleep measures and expanded measures of CMR are included.

## Strengths of the study

Our study has the major strength of introducing rural participants which allowed us to study the rural versus urban disparities in the associations between sleep health and CMR using a combination of sleep health measurements (sleep quality, sleep duration, risk of sleep apnea, and daytime sleepiness). Thus, it provides, first, a better understanding of the association between the multi-dimensions of sleep health with cardiometabolic health. Secondly, our findings add to the existing body of evidence on the need to study sleep from its multiple dimensions rather than focus on a single measure such as sleep duration or quality. Third, our large sample size gave us the power to show associations of poor sleep health with simple clinical measures of cardiometabolic risk. In this study, we see that poor sleep is associated with CMR, thus giving some evidence that targeting better sleep health in adolescents should be considered by policy makers as a strategy to prevent cardiovascular disease later in life.

## Conclusion

In conclusion, poor sleep health, assessed with multiple sleep health variables, showed a significant association with higher cardiometabolic risk in this sample of 900 Nigerian adolescents; with urban adolescents showing poorer sleep health and higher adiposity-related cardiometabolic risk than rural adolescents. Interventions to prevent CMR in adolescents should thus involve the promotion of good sleep health of adolescents across its multiple domains, having adolescents living in urban communities as important targets of such interventions. Such interventions could include information sessions by sleep experts to explain to the staff, students and parents the importance of sleep and give tips for better sleep in adolescents. On a wider scale, our recently published study on objective measures of sleep in a subsample of 170 of these 900 adolescents showed lower sleep duration and earlier wake times on school days^34^. It could be that as in other countries (USA^35^, Singapore^36^, Rwanda^37^ for example), delaying school start times in Nigeria may help increase sleep duration on school days. In turn, considering the relationship we found between lower sleep duration and worse cardiometabolic health, extending sleep opportunity and duration during the school days may have beneficial effects on cardiometabolic health^38^. Further research using longitudinal and/or experimental designs is also necessary to better understand the causal relationships underlying the association between poor sleep health and cardiometabolic risk in adolescents.

## Methods

### Study design

This was a quantitative cross-sectional study involving adolescents aged 13–19 years attending six secondary schools in a rural setting and six secondary schools in an urban setting in the Osun East Senatorial District of Osun State, Nigeria.

### Study setting

The site for the study is the Osun East Senatorial District, one of the three senatorial zones (regions) in Osun State, Nigeria, one of the most populated southern Nigerian states. We selected three Local Governmental Areas (LGAs) out of the ten LGAs in the district based on their geographical proximity and ethno-religious similarities. Thus, the communities are only different based on their rural versus urban features. The rural and urban classification is based on the categorization of the communities within the State by the State Government. Six schools were selected from 6 different rural communities (one school per rural community) since most of these communities had only one or two secondary schools. Six schools were selected for the study in Ile-Ife, the urban community, using the proportion-to-population approach.

### Study participants

The study participants were apparently healthy adolescents aged 13–19 years attending secondary schools in the selected rural and urban communities. Adolescents using sedative medications, anti-epileptics or narcotic preparations for any acute or chronic medical condition were excluded from the study using a screening questionnaire at the first school visit. We also excluded adolescents who have a known chronic medical condition.

Adolescents in the final class (senior secondary school 3 (SSS 3)) were excluded because they were taking their final examinations during the time of data collection. Finally, students living in boarding facilities within the schools were excluded due to the controlled day and night schedules in the school boarding facilities.

### Sampling procedures

The multistage sampling method was used to select study participants. First, a list of secondary schools in each community was obtained from the Education Office of the Osun State Ministry of Education. A total of 12 schools, six in the urban community and six in rural communities, were then selected using stratified sampling. At the second stage, a list of classes in each selected school was obtained, and a class was selected from each stratum of the classes from Junior Secondary School (JSS) 1 (age range 10–13 years old and 10–16 years old in urban areas and rural areas respectively) to Senior Secondary School (SSS) 2 (age range 14–16 years old, urban areas; age range 14–19 years old, rural areas) using the stratified sampling technique. In the third stage, a predetermined number of students per class were selected by simple random sampling. The number of students selected per class was proportional to the class population based on a proportionate-to-population approach.

### Background demographic information to the study

The descriptive study on sleep characteristics in rural and urban Nigerian adolescents has been published previously^25^. In summary, 900 adolescents (459 rural; 59% female; average age 15.1 ± 1.4; 49.1% of lower social class) were included in this study. We showed that there was no statistically significant difference in the sex composition of the adolescents based on their type of residence (rural or urban)^25^. The rural adolescents were older, were more in the middle and late adolescence category than early adolescence, more in the lower social class, more from polygamous family settings, and were more in the public schools. Despite being older, the rural adolescents were more likely to be in junior secondary school (vs. Senior secondary school) than the urban adolescents^25^. The urban adolescents reported significantly higher parental cardiometabolic disease (*p* < 0.001) while there was no significant difference in the physical activity levels between the rural and urban adolescents (*p* = 0.195).

### Measures

We collected the socio-demographic characteristics, sleep characteristics, and cardiovascular risk factors of the adolescents. All measures were performed in accordance with the relevant guidelines and regulations. The demographic variables included: age as at the last birthday, sex of the respondent, location of adolescents’ residence in rural or urban locality as designated by the National Population Commission. We also obtained parental history of cardio-metabolic disease by asking for history of hypertension or diabetes in the adolescents’ father and mothers. We categorized an adolescent as having a parental history of cardio-metabolic disease if either the father or the mother was known to have hypertension and/or diabetes. Further details on how information was obtained about the socio-demographic, sleep and cardiovascular variables are shown in the Table S1. The Research Assistants who assisted with the data collection were first trained during a full-day training session on the questionnaire and the data collection process by the certified Statistician and REDCap administrator. Each research participant used a tablet to administer the REDCap questionnaire for each participant in their school. Hence, the research assistant ensured that the participant understood each question and provided a reliable self-report.

### Sleep questionnaires

We obtained a self-report of the sleep characteristics of the adolescents using validated questionnaires. We measured sleep quality and sleep duration using the Pittsburgh Sleep Quality Index (PSQI), (higher scores indicate lower sleep quality, PSQI global score > 5 out of 21 indicates poor sleep quality)^39,40^, daytime sleepiness using the Epworth Sleepiness Scale for children and adolescents (higher scores indicating higher daytime sleepiness)^41^, and risk for sleep apnea with the Teen STOPBANG (risk of sleep apnea vs. low risk. Evaluate the presence of **S**noring, Ever feeling **T**ired, **O**bserved apnea, High Blood **P**ressure, **B**MI > 95th percentile, **A**cademic performance, **N**eck circumference > 95th percentile, and Male **G**ender^42^. We also used the number of categories (0–8) present in the participant as the independent variable, denominated ‘Teen STOPBANG continuous’)^42^. The Teen STOPBANG has been shown to detect obstructive sleep apnea in teens when compared to the polysomnographic cut-off of an apnoea-hypopnoea index of ≥ 1.5 events/hour, with a sensitivity of 0.64, specificity of 0.82, and a negative predictive value of 0.96^42^. The details of the characteristics of the sleep questionnaires used are provided in Table S1.

### Anthropometric measurements

The anthropometric data obtained from each adolescent included their standing height, weight, waist circumference, and neck circumference. We measured the adolescents’ standing height (cm) to the nearest 0.01 cm with a standard calibrated stadiometer, and the body weight (kg) was noted on a standard weighing scale to the nearest 0.1 kg with the adolescent dressed in minimal clothing. The body mass index (BMI) was calculated as weight (kg) divided by the square of the height (m). The waist circumference was measured with a non-elastic measuring tape using the landmark of the midway between the pubic symphysis and the xiphisternum. Also, the adolescents’ neck circumference was measured with a non-elastic measuring tape using the landmark of the mid-cervical spine and mid-anterior neck at the level of the most prominent part of the thyroid cartilage. We instructed the participants to stand with their heads held erect, eyes looking straight ahead and their necks in the horizontal plane. We measured each adolescent’s blood pressure on the right arm in the sitting position using an appropriate-sized cuff. The blood pressure was measured with the average of three readings per participant to avoid error. The age-, sex-, and height-specific percentile for BP were calculated according to WHO reference data^43,44^. We also calculated the mean arterial pressure for each participant using the standard formula. Further details on how these measurements were taken using standard guidelines are shown in Table S1.

### Random blood glucose (RBG)

We used the BGCheck BS-201 Blood Glucose Monitor (HealthCheck™) to measure the RBG of each participant. This blood glucometer utilizes the glucose dehydrogenase method. The accuracy of the glucometer is 100% (within ± 0.83mmol/L) for glucose concentrations < 5.55mmol/L and 99.7% (within ± 15% for values ≥ 5.55mmol/L).

### Cardiometabolic risk factors

These included systolic and diastolic blood pressure and calculated mean arterial pressure (MAP), BMI, waist circumference (WC), and RBG, all measured using published guidelines. We then created age- and sex-adjusted z-scores for those variables. A continuous CMR score was calculated using z-scores of mean arterial pressure (MAP), body mass index (BMI), waist circumference (WC), and random blood glucose. We calculated the WHO z-scores using previously described methods, though we used non-laboratory measures, as agreed in the permission given by the school authorities. Previously used continuous CMR scores in pediatric^45,46^ and adult populations^47^, were generated from the combination of available (i.e., non-laboratory) markers of CMR collected. A non-laboratory cardiovascular method was previously validated in the NHANES cohort in adults^48^.

### Physical activity

We assessed the physical activity (PA) of the adolescents using the Modified Physical Activity Questionnaire for Adolescents (PAQA). We asked about the frequency of the adolescents’ engaging in various physical activities in the past 7 days based on PAQA. We categorized the PA into three categories based on the cumulative frequency of PA in the past week. The three categories are Low if the score is < 4 (coded 0), Moderate if the score is 4–11 (coded 1) and vigorous if > 11 (coded 2). We used PA as an ordinal categorical variable in the models, assuming linear changes in CMR with reporting a higher level of physical activity.

### Data analysis

We analysed the data with Stata-15 (StataCorp. 2017. Stata Statistical Software: Release 15. College Station, TX: StataCorp LLC). Using the CMR score as the primary outcome variable, bivariate and multivariate analyses were used to determine the association between the CMR score and the following sleep variables: sleep duration, sleep quality, risk of sleep apnea (using the Teen STOPBANG continuous), and daytime sleepiness.

The Chi-square test was used to test the association between categorical variables, such as the rural-urban disparities in the sociodemographic characteristics of the participants and categorical variables. We used the independent t-test to compare CMR outcome continuous variables in urban vs. rural adolescents.

The univariable analysis of the relationship between CMR outcome variables and the sleep health of the adolescents was done using simple linear regression. As BMI and blood pressure were both components of the CMR score and the Teen STOPBANG continuous, we tested the association between those two variables using a Pearson’s correlation, which showed a weak significant correlation (*r* = 0.13; *p* < 0.001), probably because only blood pressure and body mass index were components of both variables. Similar weak correlations were found between the Teen STOPBANG continuous and the SBP, DBP, and MAP z scores (*r* = 0.20, *p* < 0.001; *r* = 0.11, *p* < 0.001; *r* = 0.17, *p* < 0.001), respectively.

At the multivariable level, the generalized linear model was used to identify the relationship between key z-scores of the main CMR variables (CMR score, BMI, WC, NC, MAP and RBG as outcome variables, respectively) and (1) type of residence (urban vs. rural) and (2) sleep health variables while adjusting for parental history of cardiometabolic disease and physical activity levels. In the analysis, we used the PA variable as an ordinal categorical variable (taking values 0, 1, and 2).

## Supplementary Information

Below is the link to the electronic supplementary material.

Supplementary Material 1

## Data Availability

The datasets generated during and/or analyzed during the current study are available from the corresponding author on reasonable request.
