# Supplementary material for: Poor sleep health is associated with worse cardiometabolic risk among rural and urban schooled Nigerian adolescents
Source: Sci Rep. 2025 Nov 25;15:41768. doi: 10.1038/s41598-025-25659-0 (PMC12647823; doi:10.1038/s41598-025-25659-0)
Supplement: Supplementary file 1 — Supplementary Material 1 [file 41598_2025_25659_MOESM1_ESM.docx]

## **Table S1: List of demographic variables, sleep variables and cardiometabolic risk factors measured on the adolescents.**

| **Variables** | **Measure** | **Questionnaire/Mode of assessment** | **Final measurement used and reference group (if applicable)** |
| --- | --- | --- | --- |
| **Demographic factors** | | | |
| Age | Age at last birthday | Study questionnaire | Age (years) |
| Sex | Sex of the respondent | Study questionnaire | Male vs Female  Reference group: Female |
| Location of adolescents’ residence | Rural or urban locality as designated by the National Population Commission | Study questionnaire | Rural vs Urban  Reference group: Rural |
| Parental history of cardio-metabolic disease | History of hypertension or diabetes in the adolescents’ father and mother. | We categorized an adolescent as having a parental history of cardio-metabolic disease if either the father or the mother is known to have hypertension and/or diabetes. | The parental history of cardio-metabolic disease is categorized as absent or present.  Reference group: No CMR |
| **Sleep Health Variables** | | | |
| Sleep Quality | Pittsburgh Sleep Quality Index (PSQI), | 19 questions which lead to 7 components (sleep duration, sleep onset latency, sleep efficiency, sleep quality, daytime function, sleep quality, sleep medications) each scored on a Likert scale from 0 to 3 (0: not during the past week, 1: less than once a week 2: once or twice per week, 3: 3 or more times per week)  Global score out of 21, higher scores indicate poorer sleep quality, PSQI>5 is considered poor quality sleep | PSQI global score taking values from 0-21(higher score: poorer sleep quality)^1,2^ |
| Daytime Sleepiness | Epworth Sleepiness Scale for Children and Adolescents (ESS CHAD) | Measured with the Epworth Sleepiness Scale for Children and Adolescents (ESS CHAD). It has 8 components investigating the likelihood of falling asleep in various scenarios (0: never, 1: slight chance, 2: moderate chance 3: high chance).  Global score out of 24  Higher scores indicate higher daytime sleepiness, ESS>10 indicate likelihood of a sleep disorder. | ESS CHAD global score taking values 0-24 (higher score reflects higher daytime sleepiness).^3^ |
| Risk of Sleep Apnea | Teen STOP-BANG questionnaire | Evaluates the presence of :  **S**noring  Ever feeling **T**ired  **O**bserved apnea  High blood **P**ressure  **B**MI> 95^th^ percentile  **A**cademic performance  **N**eck circumference >95^th^ percentile  Male **G**ender.^4^  If presence of 3 or more of those 8 symptoms, then the participant is at risk of sleep apnea.  Final score : risk of sleep apnea vs low risk of sleep apnea | Risk of sleep apnea or Low risk of sleep apnea  Reference group: Low risk |
| Sleep Duration | Sleep duration | This is the self-reported duration of sleep obtained from the Pittsburgh Sleep Quality Index (PSQI) questionnaire | A continuous variable reflecting hours spent asleep, as reported by the participant |
| **Cardio-metabolic Risk Measures** | | | |
| Weight | Weight of the respondent | The anthropometric data obtained by trained research assistants from each body weight (kg) was noted on a standard weighing scale to the nearest 0.1 kg with the adolescent dressed in minimal clothing. | Weight (kg) |
| Height | Height of the respondent | Adolescents’ standing height (cm) to the nearest 0.1 cm with a standard calibrated stadiometer using the standard protocol. | Height (cm) |
| Body Mass Index | Body mass index (BMI) | Calculated as weight (kg) divided by height (m) squared. | BMI (kg/m^2^) |
| Body Mass Index z-score | BMI z-score | The age- and sex-specific z-scores were calculated using the WHO Anthroplus software. | BMI z-score |
| Physical Activity (PA) | Modified Physical Activity Questionnaire for Adolescents (PAQA) | We asked about the frequency of the adolescents’ engaging in various physical activities in the past 7 days based on PAQA. | We categorized the PA into three categories based on the cumulative frequency of PA in the past week. The three categories are Low if the score is <4 (coded 0), Moderate if the score is 4-11 (coded 1) and vigorous if > 11 (coded 2).  In the analysis, we used the PA variable as an ordinal categorical variable (taking values 0, 1 and 2). We assumed a linear relationship between the different categories and the CMR variables of interest. The Beta estimate from the regression model thus represents the change in CMR variable when moving from category 0 (low PA) to category 1 (moderate PA) or from category 1 to category 2 (high PA). Moving from category 0 to category 2 thus would be represented by Beta x 2. |
| Waist circumference | Waist circumference | Measured with non-elastic measuring tape using the landmark midway between the pubic symphysis and the xiphisternum. | Waist circumference (cm) |
| Waist circumference z-score |  | The age- and sex-specific z-scores were calculated using age- and sex- specific mean (SD). Each participant’s waist circumference (WC)’s z score was calculated as follows:  (WC_participant_-mean WC_age & sex specific_)  /standard deviation WC_age & sex specific_ | Waist circumference z-score |
| Neck circumference | Neck circumference | Measured with a non-elastic measuring tape using the landmark of the mid-cervical spine and mid-anterior neck at the level of the most prominent part of the thyroid cartilage. We instructed the participants to stand with their heads held erect, eyes looking straight ahead and their necks in the horizontal plane. | Neck circumference (cm) |
| Neck circumference (NC) z-score |  | The age- and sex-specific z-scores were calculated using age- and sex- specific mean (SD). Each participant’s neck circumference’s z score was calculated as follows:  (NC_participant_- mean NC_age & sex specific_) /standard deviation of NC_age&sex specific_ | Neck circumference z-score |
| Blood pressure | Systolic and Diastolic Blood pressure (SBP and DBP) of the respondents | Measured on the right arm in the sitting position using an appropriate-sized cuff. We used the average blood pressure out of the measured three readings per participant. | SBP (mm Hg)  DBP (mm Hg) |
| DBP z-score and SBP z-score |  | The age-, sex-, and height-specific percentile for DBP and SBP were calculated according to WHO reference data.^5^ | DBP z-score  SBP z-score |
| Mean Arterial Pressure | Mean Arterial Pressure  (MAP) of the respondents | MAP = DBP + 1/3(SBP – DBP) | MAP (mm Hg) |
| MAP z-score | determined the mean and standard deviation of MAP for the whole sample to calculate the MAP z-score | (MAP_participant_- mean MAP_sample_)/ standard deviation of MAP_sample_ | MAP z-score |
| Random blood glucose (RBG) | Glucometer-derived RBG | We used a glucometer to measure the RBG of each participant. | Plasma glucose concentration (mmol/L) |
| RBG z-score |  | Each participant’s RBG z score was calculated as follows: (RBG_participant_- mean RBG_sample_)/ standard deviation of RBG_sample_ | RBG z-score |
| CMR score | Continuous CMR score | Generated from the combination of four CMR measures namely:  i) blood pressure (BP) = MAP z-score , ii) body mass index (BMI) z-score, iii) waist circumference (WC) z-score, iv) random blood glucose (RBG) z-score.^6^ We used these simple non-laboratory measures based on earlier reports of the use of non-laboratory based risk prediction methods for cardiovascular risk being as accurate as the methods requiring laboratory information.^7^ | We calculated the continuous CMR score by obtaining the mean of the z-scores of the CMR factors such that each factor was equally weighted.  CMR score = (MAP z-score+BMI z-score+ WC z-score + RBG z-score)/4 |

1. Buysse DJ, Reynolds CF, Monk TH, Berman SR, Kupfer DJ. The Pittsburgh Sleep Quality Index: a new instrument for psychiatric practice and research. Psychiatry Res. 1989;28:193–213.

2. Aloba OO, Adewuya AO, Ola BA, Mapayi BM. Validity of the Pittsburgh Sleep Quality Index ( PSQI ) among Nigerian university students. Sleep Med. 2007;8:266–70.

3. Janssen KC, Phillipson S, O’connor J, Johns MW. Validation of the Epworth Sleepiness Scale for Children and Adolescents using Rasch analysis. Sleep Med [Internet]. 2017 [cited 2022 Nov 10];33:30–5. Available from: http://dx.doi.org/10.1016/j.sleep.2017.01.014

4. Combs D, Goodwin JL, Quan SF, Morgan WJ, Parthasarathy S. Modified STOP-Bang Tool for Stratifying Obstructive Sleep Apnea Risk in Adolescent Children. 2015;4:1–11.

5. Falkner B, Daniels SR. National High Blood Pressure Education Program Working Group on High Blood Pressure in Children and Adolescents. The fourth report on the diagnosis, evaluation, and treatment of high blood pressure in children and adolescents. Vol. 44, Hypertension. 2004. p. 114(2 Suppl 4th Report):555-76.

6. Roche J, Corgosinho FC, Isacco L, Scheuermaier K, Pereira B, Gillet V, et al. A multidisciplinary weight loss intervention in obese adolescents with and without sleep-disordered breathing improves cardiometabolic health, whether SDB was normalized or not. Sleep Med [Internet]. 2020; Available from: https://doi.org/10.1016/j.sleep.2020.06.030

7. Gaziano TA, Young CR, Fitzmaurice G, Atwood S, Gaziano JM. Laboratory-based versus non-laboratory-based method for assessment of cardiovascular disease risk: the NHANES I Follow-up Study cohort. Lancet. 2008;371(9616):923–31.
